# The Effect of Fucoidan on Cellular Oxidative Stress and the CatD-Bax Signaling Axis in MN9D Cells Damaged by 1-Methyl-4-Phenypyridinium

**DOI:** 10.3389/fnagi.2018.00429

**Published:** 2019-01-16

**Authors:** Zhigang Liang, Zhuli Liu, Xuwen Sun, Manli Tao, Xiao Xiao, Guoping Yu, Xiaomin Wang

**Affiliations:** ^1^Department of Neurology, Yantai Yuhuangding Hospital Affiliated to Qingdao University, Yantai, China; ^2^Brain Major Disease Research Institute, Capital Medical University, Beijing, China

**Keywords:** fucoidan, MN9D cells, 1-methyl-4-phenyl pyridine, oxidative stress, lysosome, apoptosis

## Abstract

**Background:** The purpose of this study was to investigate the impact of fucoidan (FUC) on the oxidative stress response and lysosomal apoptotic pathways in the Parkinson disease (PD) cell model.

**Methods:** The Dopaminergic nerve precursor cell line(MN9D) cells that served as a PD model in this study underwent damage induced by 100 μM 1-methyl-4-phenyl pyridine (MPP^+^). Cell viability was assessed after FUC treatment and intracellular SOD GSH was measured via immunofluorescence assay. Cellular changes in cathepsin D, Autophagy marker Light Chain 3-II (LC3-II), and apoptotic protein Bax were assessed by Western blot. The expression of Cat D, LC3-II, and B cell lymphoma-2-associated x protein (Bax) was also measured after addition of the cathepsin inhibitor, pepstatin A.

**Results:** The results indicated that MN9D cell viability decreased by 50% within 24 h after 100 μM MPP^+^ induced toxicity. Pretreatment with 100 μM Fucoidan reduced cellular expression of LC3-II and CatD in 3 h and suppressed the induction of Bax protein. After pepstatin A treatment, Bax expression was significantly downregulated.FUC reversed the reduction of superoxide dismutase (SOD) L-Glutathione(GSH), decreased cell viability, and apoptosis induced by MPP^+^ in 6 h, suggesting that Fucoidan can attenuate damage to MN9D cells induced by MPP^+^.

**Conclusions:** Fucoidan protected lysosomes, reduced the expression of LC3-II, inhibited the expression of CatD-Bax and the oxidative stress response, suppressed apoptosis, and thus conferred protective effects for dopaminergic neural cells. FUC may have neuroprotective effects on PD and further research is needed.

## Highlights

In the study, we found that Fucoidan can attenuate damage to MN9D cells induced by MPP^+^. Fucoidan reduced cellular expression of LC3-II and CatD and suppressed the induction of Bax protein. FUC reversed the reduction of SOD GSH, decreased cell viability, and apoptosis induced by MPP^+^.

## Introduction

Parkinson's disease (PD) is a common neurodegenerative disease with evolving layers of complexity. It has long been characterized by classical motor features of Parkinsonism associated with Lewy bodies and loss of dopaminergic neurons in the substantia nigra, but the mechanisms underlying its pathogenesis remain unclear (Thomas and Beal, 2007). The oxidative stress response and mitochondria-mediated neuronal apoptosis play critical roles in the initiation and development of degenerative disease. Cathepsin D (CatD) is involved in mitochondria-regulated apoptosis, and its role in this process has become a hotspot in cell death research worldwide. Studies using MPTP to produce rhesus PD models observed up-regulation of cathepsin D in the caudate nucleus and an increase in the number of lysosomes in neurons of that brain region. While overexpression of cathepsin D in neuroblastoma cell lines promoted apoptosis (Yelamanchili et al., 2011), studies have found that glycosaminoglycan (GAG) negatively regulates oxidative-stress-induced cell apoptosis at the lysosomal and mitochondrial levels (Dudas et al., 2008). Glycosaminoglycans have been shown to inhibit lysosomal cathepsin activities. GAG-mimetics reduce intracellular ROS levels and inhibit mitochondrial membrane potential collapse, cytochrome c release, and caspases-9 and−3 activation without affecting the extrinsic apoptotic pathway (Yue et al., 2009). Fucoidan (FUC) is a polysaccharide extracted from brown seaweed (Supplementary Figure 1). The FUC used in this study is a standard sample purchased from China National Institute for the Control of Pharmaceutical and Biological Products (16121901). The main components are: L-fucose-4-sulfate, purity 98%, average molecular weight 189KD,Dissolved in water. It has antioxidant, anticoagulant, and anti-aging effects. Studies have shown that FUC also plays a neuroprotective role in the AD model (Barbosa et al., 2014). Previous studies reported that FUC can protect the cell model of PD and the mouse model of MPTP damage by anti-oxidative stress. However, there has been no further study on the mechanism of FUC protection against PD (Luo et al., 2009). We used 1-methyl-4-phenyl pyridine ion (MPP^+^) to damage a dopaminergic cell line of MN9D cells. The function of FUC, its relationship to oxidative damage, and the Cathepsin D-Bax signaling axis were examined in this PD cell model. Selegilin (SEL), an irreversible monoamine oxidase-B (MAO-B) inhibitor used to help control the symptoms of Parkinson's disease, served as a positive control reagent at an effective concentration of 10 μM (Riederer and Laux, 2011). The purpose of this experiment was to elucidate the mechanism underlying the intervention of FUC in oxidative stress-induced neuronal apoptosis and associated potential targets.

## Materials and Methods

### Cells, Materials, and Experimental Groups

MN9D cells were purchased from the cell compartment of the Chinese Academy of Medical Sciences and stored in our laboratory. The oxidative stress index and cell morphology were detected in the control group, MPP^+^ group, FUC preconditioning group, and selegiline preconditioning group. Protein expression via Western blot was detected in the control group, MPP^+^ group, FUC preconditioning group, selegiline preconditioning group, and CatD inhibitor group (pepstatin A group).

### Establishment of a PD Cell Model and Experimental Grouping

The supernatant was removed by centrifugation at 1,000 rpm for 5 min. The pellet was suspended in DMEM/F12 [GIBCO BRL (Gaithersburg, MD, USA)] +10% Newborn calf serum NCS medium [PAA (Linz, *Austria*)]. The cell suspension was aspirated, diluted with appropriate culture medium, and transferred into a sterile culture flask at 37°C, in 5% CO_2_ cell culture medium. Culture medium was replaced the next day. Cells were cultured 48 h into the exponential growth period after subculture. Cells were seeded in Poly-L-lysine-coated 96-well plates at a density of 1 × 10^5^/ml and at a volume of 100 μl per well. As in our previous study (Lehri-Boufala et al., 2015), 100 μmol/L MPP^+^ (Sigma U.S.) was used to damage the MN9D cells for 24 h to prepare the PD cell model, hence establishing the model and control groups. After cells were stabilized for 24 h, DMEM/F12 medium containing 5% NCS was replaced. Control Groups were seeded with the same volume of saline. After cells were stabilized for 24 h, DMEM/F12 medium containing 5% NCS was replaced with 1 × 10^−8^, 10^−7^ - 10^−4^ μmol /L FUC medium for 1 h. One hundred micromole per liter MPP^+^ was added for 24 h, and the optimal protection concentration (100 μmol/L) of FUC was determined by detection of cell viability. MN9D cells were seeded in Poly-L-lysine-coated 6-well plates at a density of 1 × 10^5^/ml and a 0.5 ml volume per well. After incubation for 24 h, cells were cultured in DMEM/F12 medium supplemented with 5% NCS and 100 μmol/L FUC, 10 μmol/L pepstatinA (SIGMA St. Louis, Missouri, USA), respectively. Activity of CatD (Biovision, Palo Alto, USA) was detected by fluorometer after 3 and 6 h of MPP^+^ incubation in 100 μmol/L, respectively. The expression of CatD protein and Bax Bcl-2 protein [Sigma (St. Louis, Missouri, USA)] was detected by Western blotting after 3 and 6 h of MPP^+^ incubation in 100 μmol/L, respectively. Antibody and GAPDH (mouse monoclonal anti-tyrosine hydroxylase, ascites fluid) were obtained from Sigma (St. Louis, Missouri, USA). All other reagents were of analytical grade and were obtained from SIGMA (St. Louis, Missouri, USA). Twenty-four hours morphological changes were observed.

### FUC and Cell Viability of MN9D, Intracellular SOD, and GSH in MPP^+^-Treated MN9D Cells

The cell survival rate was measured by MTS kit [Promage (U.S.)]. MN9D cells were inoculated into Poly-L-lysine-coated 96-well plates. After treatment with each group of drugs, DMEM/F12 medium containing 5% NCS was replaced. After incubation for 1 h, the optical density (OD) was measured by a microplate reader. The OD value of the MPP^+^ group and different concentrations of the FUC group/OD values of the control group were analyzed for group comparison.

MN9D cells were seeded in Poly-L-lysine-coated 6-well plates at a density of 1 × 10^5^/ml and a 0.5 ml volume per well. The activity of SOD and GSH [Cell Biolabs (California, USA)] in the MN9D cells was measured by fluorometer, and the SOD GSH activity of the cells was measured according to the procedure of kit operation (the activity unit is U/L).Morphological changes of cells were observed via inverted phase contrast microscopy. The cells were randomly divided into 4 fields and observed under 100 magnifications.

Cathepsin D activity can be quantified by relative fluorescence units (RFU) per million cells. MN9D cells were seeded onto a 96-well plate in a Poly-L-lysine-coated, light-tight, inoculation volume of 0.5 ml/well at a density of 1 x 10^5^/ml. The activity of CatD in the MN9D cells was measured by fluorometer (RFU/10^6^ cells) according to the instructions of the Biovision CatD Activity Kit for 3–6 h after each treatment.

### Western Blot Analysis in MN9D Cells

Levels of CatD protein, LC3-II protein and Bax in MN9D cells were detected by Western-blot. A volume of 99 μl protein lysis buffer was added to a 6-well plate, along with 1 μl of protease inhibitor cocktails, and these were kept on ice for 5 min. A cell scraper was used to collect the cell lysate, which was transferred to an Epperdorf tube. The lysate was sonicated three times, kept in an ice bath for 30 min, and centrifuged at 12,000 rpm at 4°C for 5 min. The supernatant was transferred to another Eppendorf tube and the pellet was discarded. Protein was denatured at 95°C for 5 min and stored at −20°C. Separating (10–12%) and stacking gels (5%) were prepared. Samples were loaded into the gel, and the electrophoresis tank was filled with buffer. After loading 60 μg samples, the electrophoresis ran at 80 V for 20 min and was changed to 120 V for 80–100 min until the bromophenol blue reached the bottom of the separation gel. The gel was then removed from the electrophoresis apparatus. The cassette was placed with the black side down into the tray with transfer buffer. Items were placed on the black side of the cassette in the following order: sponge pad, 3 layers of filter paper, gel, nitrocellulose membrane, 3 additional layers of filter paper, and an additional sponge pad. Air bubbles were removed, and the cassette was firmly closed and placed in the electrode module. It was transferred with 100 V for 60 min.

Upon completion of the transfer, the blotting sandwich was disassembled and the membrane was removed. The membrane was washed with PBS for 10 min, and blotted with 5% milk for 1–2 h by rocking at room temperature. Mouse primary antibodies; anti-Bax (1:500), anti-CatD (1:2,000) and anti-LC3-II (1:2,000) as well as internal control anti-GAPDH (1:10,000) were added and the samples were allowed to incubate overnight at 4°C and for another 30 min at room temperature the next day. The membrane was washed 3 times with PBST for 10 min each time. IRDye 800 (1:10,000)-labeled goat anti-mouse secondary antibody was incubated with the membrane at room temperature for 1 h. The membrane was again washed 3 times with PBST for 10 min each time. The residual Tween-20 on the membrane was washed off with PBS and the membrane was scanned with an Odyssey infrared imaging system. The Odyssey infrared imaging system was used to scan the OD value of the protein strip, and the levels of CatD and Bax were measured. The OD value of the control group was quantified.

### Data Analysis and Statistical Tests

Statistical analysis was performed using Prism 5.0. The difference between multiple sets of data was analyzed with ANOVA followed by the Tukey *post-hoc* test. Results are given as mean ± standard error (Mean ± s.e.m), with *P* < 0.05 indicating statistical significance.

## Results

### Protective Effect of FUC on the Viability and Morphology of MN9D Cells After MPP^+^-Damaged

**Figure 1:**
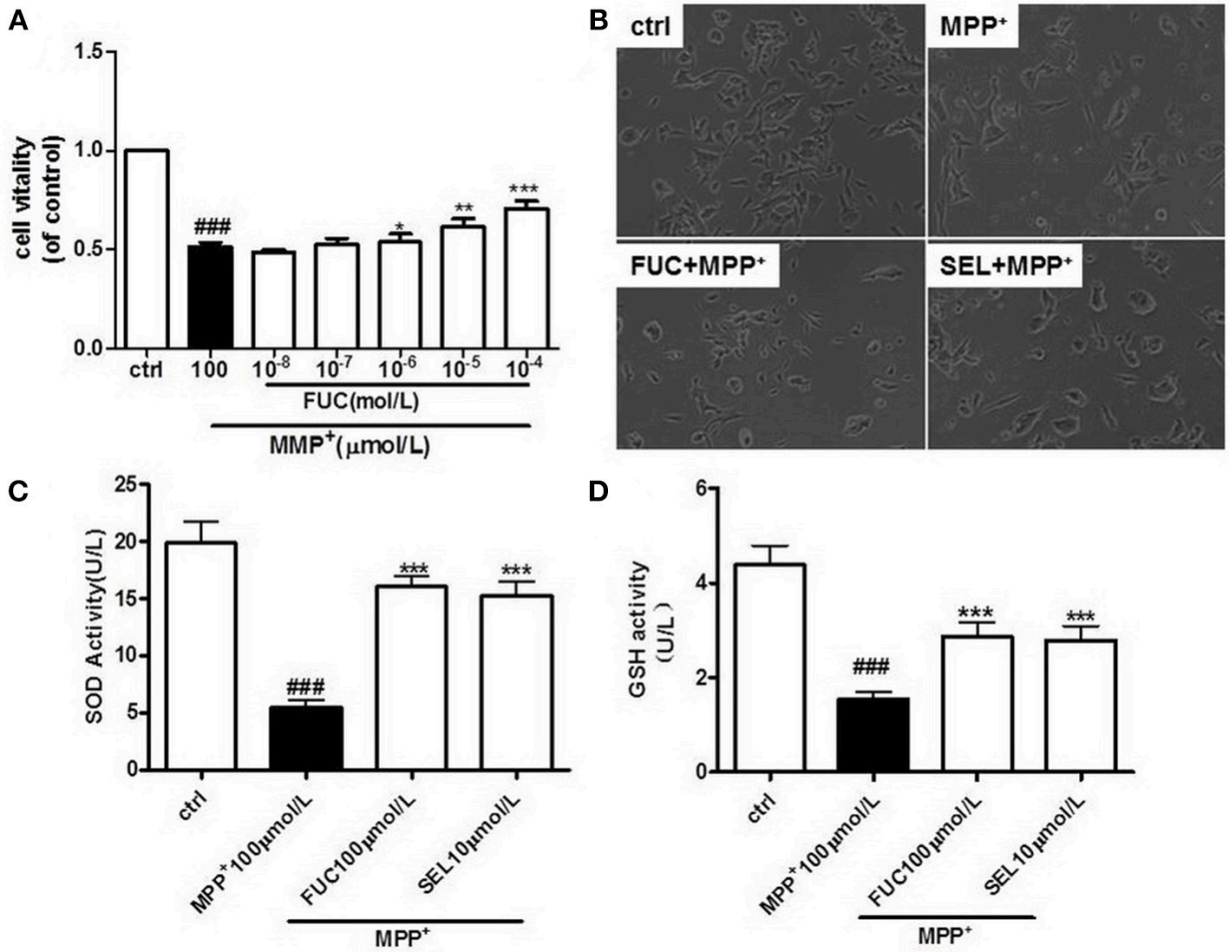
FUC protects the activity and morphology of MN9D dopaminergic neurons damaged by MPP^+^. **(A)** MN9D cells were pretreated with different concentrations of FUC for 1 h followed by co-treatment with 100 μM MPP^+^ for 24 h. Cell vitality was detected by MTS assay. **(B)** The morphology of MN9D cell in each group,damaged by MPP^+^ for 12h.(Magnification: 100 ×). **(C)** The intracellular SOD activity of MN9D cell model. **(D)** The intracellular GSH activity of MN9D cell model. Ctrl (without MPP^+^ and FUC); MPP^+^ (MPP^+^ only); FUC100μM+ MPP^+^; SEL10μM + MPP^+^. The results are means ± SEM, *n* = 6. ^*^P < 0.05; ^**^P < 0.01; ^***^P < 0.001 vs. MPP^+^-treated cures, ^###^*P* < 0.001 vs. control.

To determine the effective protective concentration of FUC for MN9D cells, MN9D cells were pretreated at a gradient of 1 × 10^−8^ M to 10^−3^ M FUC over 24 h. Cell viability was measured using an MTS kit. Treatment of MN9D cells with FUC alone for 24 h unsignificantly affected cell viability (Supplementary Figure 1). As shown in Figure 1A, 1 × 10^−6^ M to 10^−4^ M FUC effectively inhibited MPP^+^-induced damage in MN9D cells. Cell viability was higher than in MPP+ treated cells. Among them, 100 μM FUC significantly increased cell viability. For this reason, 100 μM was chosen as the effective treatment concentration (*P* < 0.001). MN9D cells were then pretreated with 100 μM of FUC or SEL (10 μM) as a positive control, followed by 100 μM MPP^+^ for another 24 h. Cell morphology and number was evaluated under a microscope. Results indicated that, after 24 h of treatment with 100 μM MPP^+^, there were significantly fewer MN9D cells, and they were smaller in size. Cell protrusions shortened (Figure 1B). The FUC pretreatment group cell processes shortened, and the number of cells increased compared with the MPP^+^ group (Figure 1B); SEL also increased MN9D cell number (Figure 1B).

### FUC Increases Intracellular SOD and GSH in PD Cells Model

MN9D cells were pretreated with 100 μM FUC for 1 h, followed by 100 μM MPP^+^ for another 12 h. Intracellular SOD and GSH activity was detected using a fluorometer, according to experimental procedures in SOD and the GSH test kit. SOD and GSH were significantly lower than in control cells after MPP^+^ treatment, which suggested that neurotoxicity induced by MPP^+^ decreased cellular antioxidant capacity. However, pretreatment with 100 μM FUC rendered intracellular SOD and GSH levels to be higher than in MPP^+^ treated cells. These results suggested that FUC increased SOD and GSH synthesis in the PD cell model. Treatment with 10 μM SEL also increased intracellular SOD and GSH (Figures 1C,D).

### FUC Causes Downregulation of CatD Activity in PD Model MN9D Cells

**Figure 2:**
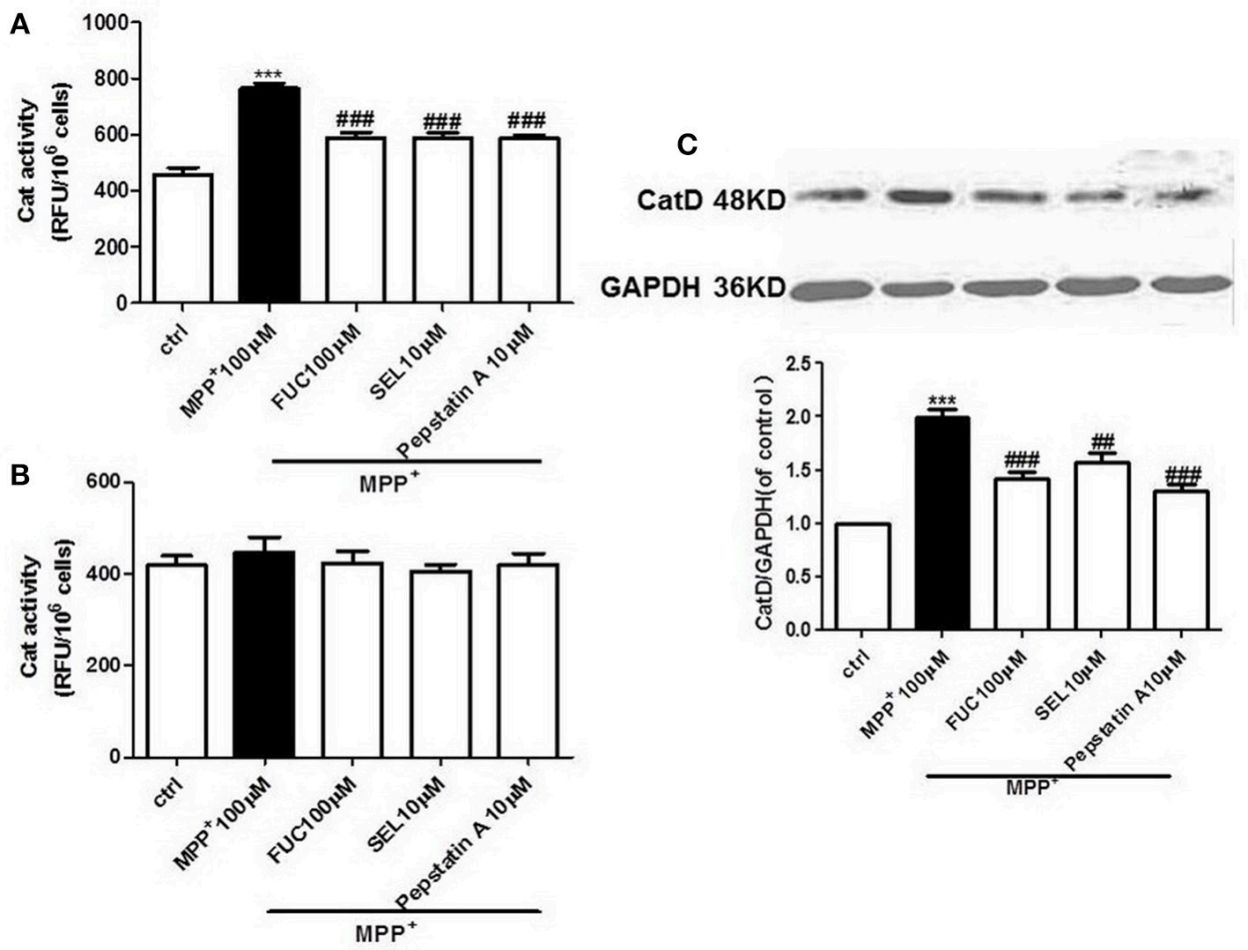
The effect of FUC on the activity of CatD and the expression of CatD in MN9D cells damaged by MPP^+^. **(A)** The effect of FUC on CatD activity in MN9D cells model in 3 h. **(B)** The effect of FUC on CatD activity in MN9D cells Model in 6 h (CatD activity was detected by fluorometer). **(C)** The expression CatD in MN9D cells damaged by MPP^+^ in 3 h (western blot). Ctrl; MPP^+^ (MPP^+^μM) only; FUC (100 μM) + MPP^+^; SEL10 μM + MPP^+^; CatD inhibitor (pepstatinA 10 μM + MPP^+^). The results are means ± SEM, *n* = 6. ^**^P < 0.01; ^***^P < 0.001 vs. control, ^##^*P* < 0.01; ^###^*P* < 0.001 vs. MPP^+^ treated cures.

MN9D cells were pretreated with 100 μM FUC for 1 h, followed by 100 μM MPP^+^ treatment for 3-6 h. CatD activity was detected by fluorometer (RFU/10^6^ cells). Results revealed that 3 h treatment with 100 μM MPP^+^ upregulated CatD activity, and 100 μM FUC antagonized MPP^+^ induced cell damage and downregulated CatD activity. SEL, which served as a positive control, also downregulated CatD activity. The CatD inhibitor pepstatin A (10 μM) significantly downregulated CatD activity. To conclude, FUC reduced MPP^+^-induced CatD activity, in MN9D cells (Figure 2A). Results revealed that the activity of CatD in each group had no significant change after 6 h (Figure 2B).

### FUC Causes Downregulation of CatD Expression in PD Model MN9D Cells

MN9D cells were pretreated with 1 × 10^−5^ M FUC for 1 h, followed by 100 μM MPP^+^ treatment for 3 h. Cell lysate was extracted and CatD protein levels were detected by Western blot. Results showed that 3 h of treatment with 100 μM MPP^+^ upregulated CatD protein expression, while 100 μM FUC mitigated MPP^+^-induced cell damage and downregulated CatD expression. SEL also downregulated CatD expression. In addition, the CatD inhibitor pepstatinA (10 μM) significantly downregulated CatD expression (Figure 2C).

### FUC Reduced the Expression of Apoptotic Protein Bax in PD Model MN9D Cells

**Figure 3:**
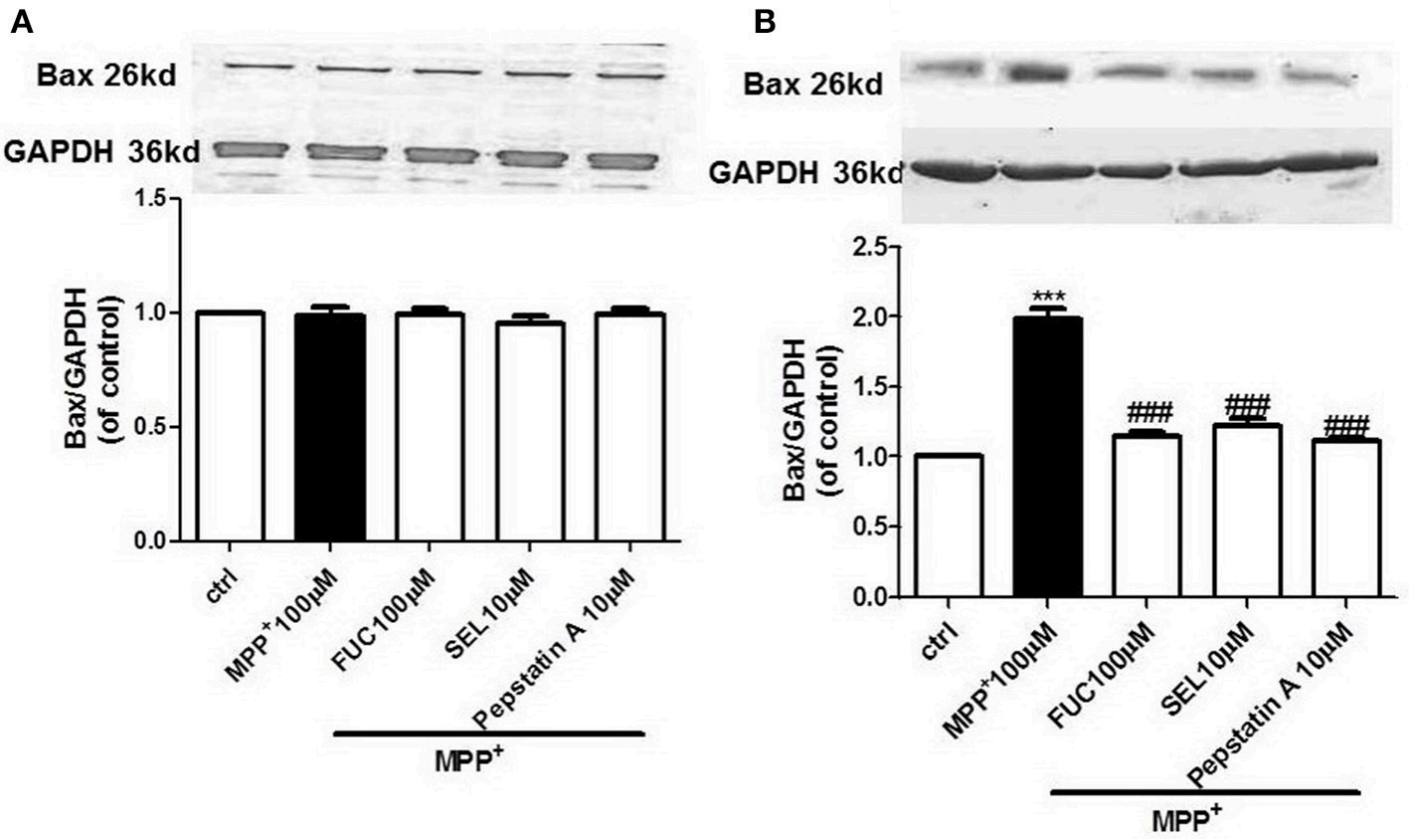
The effect of FUC on the expression of Bax protein in MN9D cells. **(A)** The effect of FUC on the expression of Bax in MN9D cells damaged by MPP^+^ at 3 h (western blot). **(B)** The effect of FUC on the expression of Bax in MN9D cells damaged by MPP^+^ at 6 h (western blot). ctrl; MPP^+^ (MPP^+^ 100 μM only); FUC (100 μM) + MPP^+^; SEL10 μM + MPP^+^; CatD inhibitor pepstatinA [pepstatinA (10 μM)+ MPP^+^]. The results are means ± SEM, *n* = 6. ^**^P < 0.01; ^***^P < 0.001 vs. control, ^###^*P* < 0.001 vs. MPP^+^ treated cures.

MN9D cells were pretreated with 100 μM FUC for 1 h, followed by 100 μM MPP^+^ treatment for 3–6 h. Cell lysate was extracted and Bax protein levels were detected by Western blot. Results revealed that the expression of Bax protein in each group had no significant changes after 3 h (Figure 3A). Results also revealed that 6 h of treatment with 100 μM MPP^+^ upregulated Bax protein expression (Figure 3B), while 100 μM FUC antagonized MPP^+^-induced cell damage and downregulated Bax expression. SEL also downregulated Bax expression and served as a positive control. The CatD inhibitor pepstatinA (10 μM) significantly downregulated expression of both CatD and Bax.

### FUC Causes Downregulation of LC3-II Expression in PD Model MN9D Cells

**Figure 4:**
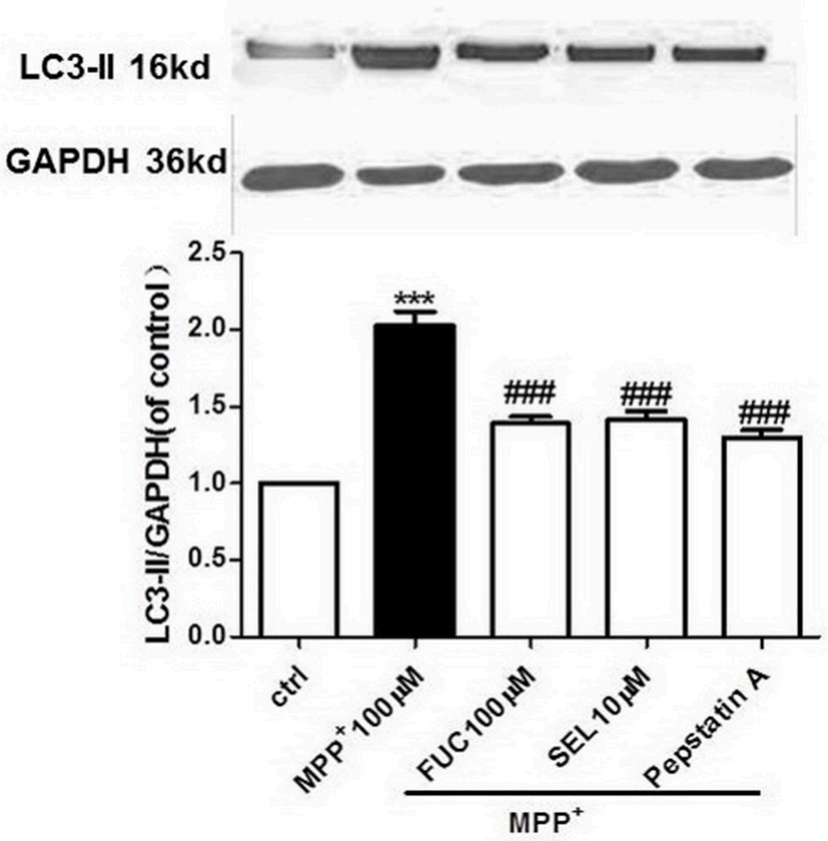
The effect of FUC on the expression of LC3-II protein in MN9D cells damaged by MPP^+^ 3 h (western blot). ctrl; MPP^+^ (MPP^+^ 100 μM only); FUC(100 μM) + MPP^+^; SEL10 μM + MPP^+^; CatD inhibitor pepstatinA [pepstatinA (10 μM)+ MPP^+^]. The results are means ± SEM, *n* = 6. ^**^P < 0.01; ^***^P < 0.001 vs. control, ^###^*P* < 0.001 vs. MPP^+^ treated cures.

MN9D cells were pretreated with 100 μM FUC for 1 h, followed by 100 μM MPP^+^ treatment for 3 h. Cell lysate was extracted and LC3-II protein levels were detected by Western blot analysis. Results revealed that a 3 h treatment with 100 μM MPP^+^ upregulated LC3-II protein expression, and 100 μM FUC antagonized MPP^+^ induced cell damage, downregulating LC3-II expression. SEL, which served as a positive control here, also downregulated LC3-II expression. The CatD inhibitor pepstatin A (10 μM) significantly downregulated LC3-II expression. To conclude, FUC reduced MPP^+^-induced LC3-II expression in MN9D cells (Figure 4).

## Discussion

Parkinson's disease (PD) is the second most common degenerative disease of the nervous system (Thomas and Beal, 2007). The major pathophysiological changes include loss or degeneration of dopaminergic neurons in the substantia nigra of the midbrain, which reduces levels of dopamine in the striatum and results in extrapyramidal symptoms. The etiology of PD has not yet been elucidated and this disease may be due to interplay between multiple factors (Riederer and Laux, 2011; Harris et al., 2012). At present, the treatment of Parkinson's disease involves mainly symptomatic therapy affecting the dopamine metabolic pathway (AlDakheel et al., 2014), and cannot delay disease progression. Therefore, finding a neuroprotective drug has become a new focus in the realm of PD research. Our study found that pretreatment with an FUC gradient concentration could increase the survival rate of MN9D cells at 100 μM (*P* < 0.001). The cell survival rate of FUC preconditioning group and SEL preconditioning group was significantly higher than that of the MPP^+^ group (*P* < 0.001). Compared with the control group, the levels of SOD and GSH in the MPP^+^ group were significantly lower than those in the MPP^+^ groups (*P* < 0.01). The level of CatD, LC3-II protein in MPP^+^ group increased significantly at 3 h, and decreased significantly in the FUC preconditioning and SEL preconditioning groups (*P* < 0.001). The level of CatD protein or CatD activity in MPP^+^ group did not increase significantly at 6 h when compared with the MPP^+^ group; Bax level decreased significantly in the FUC preconditioning and SEL preconditioning groups at 6 h (*P* < 0.001). CatD activity and CatD protein, LC3-II, and Bax expression in the pepstatin group was significantly decreased compared with that in the pepstatin A group (*P* < 0.05). The FUC group was similar to pepstatin A group, suggesting that FUC may have an inhibitory effect on CatD.

Oxidative stress may be the most common mechanism underlying PD pathogenesis (Kim et al., 2015). Autopsies of brains of PD patients have shown extensive oxidative stress in the substantia nigra, including increases in free iron ions, GSH reduction, dysfunctional mitochondrial complex I, and large numbers of damaged lipids, proteins, and DNA. 1-methyl-4-phenyl-1,2,3,6-tetrahydropyridine (MPTP), 6-hydroxydopamine (6-OHDA), and other neurotoxins can also generate free radicals and impair striatal DA neurons, all of which suggests that oxidative stress is closely involved in the initiation and development of PD (Uttara et al., 2009). Autophagy is a lysosome-dependent protein degradation pathway (Damme et al., 2015), and it can lead to protein aggregation or even the degradation of a whole organelle, and is considered to be closely related to PD (Xilouri and Stefanis, 2011; Nah et al., 2015). Studies have shown that autophagy might be a trigger for apoptosis. An increase in LC3-II indicates upregulated autophagy and the onset of cell apoptosis (Ghavami et al., 2014). Recent studies have shown that autophagy may lead to PD (Radad et al., 2015). MPP^+^ causes mitochondrial dysfunction and oxidative stress, which eventually leads to cell death. MPP^+^ can thus be used to generate a cellular model of a PD cell (Keane et al., 2015). Most recent studies have shown that MPP^+^ leads to mitochondrial dysfunction via the lysosomal pathway. Lysosomes release CatD, which leads to further cell apoptosis. Our study found that LC3-II protein expression became significantly higher in MN9D cells after 3 h of treatment with MPP^+^. Pretreatment with FUC reduced LC3-II expression, confirming that FUC alleviated MPP^+^-induced lysosomal damage in MN9D cells.

Polysaccharides are important macromolecules *in vivo*. They provide scaffolds and serve as an energy supply (Park et al., 2013). Studies have shown that glycosaminoglycans (GAG) negatively regulate cell apoptosis induced by oxidative stress at both the lysosome and mitochondria levels (Lehri-Boufala et al., 2015). FUC is very similar to a GAG in structure (Wang et al., 2016). Previous studies have also shown that FUC has antioxidant and anticoagulant effects, as well as an antioxidant neuroprotective effect in the cellular model of AD (Jhamandas et al., 2005). Recent research indicated that FUC has a protective function for the PD cell model via anti-inflammation (Cui et al., 2012; Zhang et al., 2014). Our work builds upon previous findings by investigating the effect of FUC on the antioxidant index and lysosome-associated protein in MN9D cell models of PD induced by MPP^+^. Results revealed that FUC antagonized cell death induced by MPP^+^ effectively in a dose-dependent manner. Results also showed that 100 μM fucoidan counteracted neurotoxicity induced by MPP^+^, reduced LC3-II and CatD expression and protected mitochondrial membrane potential. FUC also reversed the reduction of SOD and GSH in MN9D cells caused by 6 h of MPP^+^ treatment, and downregulated Bax expression, thus increasing cell viability and preserving MN9D cell morphology. FUC reduced the concentration of oxidant free radicals and inhibited the expression of apoptotic protein Bax through the mitochondrial and lysosomal pathway, which eventually prevented MN9D from being damaged by MPP^+^.

Our findings suggest that FUC protects MN9D in the PD model by decreasing LC3-II and CatD expression, oxidative stress-induced cell damage, and Bax expression. FUC also increases cell viability and preserves cell morphology. In this study, we did not further detect the mRNA of Bax, CatD, and LC-II, and did not detect more apoptotic factors. We also did not verify the efficacy of FUC on other PD animal models. In the future, we will further explore the protective effects and related targets of more PD cell models and animal models. Studies have shown that selegiline has neuroprotective effects, countering oxidative stress and excitatory amino acid toxicity, as well as increasing neurotrophic factors. Selegiline can improve symptom volatility, reduce the amount of L-DA, but its impact on sleep, blood pressure and other side effects when taken for a long time (The Parkinson Study Group, 1993; Mytilineou et al., 1997). However, FUC as a marine plant-derived drugs, has fewer side effects than currently utilized treatments.

## Conclusions

In summary, lysosome-associated CatD-Bax and oxidative stress may be the mechanisms associated with FUC action in the PD model. FUC may have neuroprotective effects on PD and further research is needed.

## Author Contributions

ZhiL and XW designed the experiment and provided funding. XX and MT made the cell culture and morphological examinations. ZhuL and XX made molecular biology experiments. GY and ZhiL made the experimental data statistics. ZhiL and ZhuL wrote the article.

### Conflict of Interest Statement

The authors declare that the research was conducted in the absence of any commercial or financial relationships that could be construed as a potential conflict of interest.

## Supplementary Material

Supplementary Figure 1 **(A)** The chemical structure of FUC. **(B)** The effect of FUC on MN9D Cell vitality.
