# Supplementary figures and images for: The Effect of Fucoidan on Cellular Oxidative Stress and the CatD-Bax Signaling Axis in MN9D Cells Damaged by 1-Methyl-4-Phenypyridinium
Source: Front Aging Neurosci. 2019 Jan 16;10:429. doi: 10.3389/fnagi.2018.00429 (PMC6343539; doi:10.3389/fnagi.2018.00429)

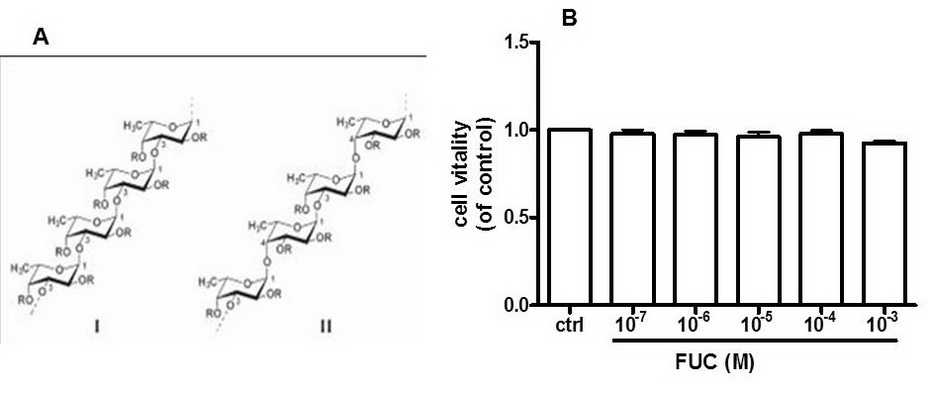

Supplement: Supplementary Figure 1 — (A) The chemical structure of FUC. (B) The effect of FUC on MN9D Cell vitality. [file Image_1.JPEG]
